# Parents’ Own Health-Related Experiences of a Weighted Blanket Intervention for Children with ADHD and Sleep Problems: A Mixed Methods Study

**DOI:** 10.3390/ejihpe16050057

**Published:** 2026-04-23

**Authors:** Julia S. Malmborg, Petra Svedberg, Jens Nygren, Håkan Jarbin, Ingrid Larsson

**Affiliations:** 1 School of Health and Welfare, Halmstad University, SE-301 18 Halmstad, Sweden; julia.soderstrom_malmborg@hh.se (J.S.M.); petra.svedberg@hh.se (P.S.); jens.nygren@hh.se (J.N.); 2 Child and Adolescent Psychiatry, Department of Clinical Sciences, Lund University, SE-221 84 Lund, Sweden; hakan.jarbin@regionhalland.se; 3 Child and Adolescent Psychiatry, Region Halland, SE-301 85 Halmstad, Sweden

**Keywords:** attention-deficit/hyperactivity disorder, convergent mixed methods, experiences, health status, parent, sleep problems, sleep intervention, well-being

## Abstract

Background: Parents of children with attention-deficit/hyperactivity disorder (ADHD) and sleep problems can experience challenges and negative health effects. The aim of this study was to explore parents’ own health-related experiences as their child with ADHD and sleep problems underwent a sleep intervention with a weighted blanket. Methods: A convergent mixed methods design was undertaken. Sociodemographic and questionnaire data were collected from 68 parents at baseline and at the 16-week follow-up. Paired-samples *t*-tests were used to analyze the data. An inductive qualitative content analysis was used to analyze interviews with 21 parents after the follow-up. An integrative analysis was performed and assessed for confirmation, expansion, or disconfirmation. Results: At the follow-up, parents reported improvements in their own health status (EQ-5D-3L—index 0.83 ± 0.15 vs. 0.87 ± 0.13; *p* = 0.034), in well-being (Outcome Rating Scale—individual 7.08 ± 2.22 vs. 7.55 ± 1.82; *p* = 0.045), and in family life (the Brief Child and Family Phone Interview—family comfort score 5.62 ± 1.62 vs. 5.14 ± 1.66; *p* = 0.003). Parents’ health-related experiences were described as: (1) having a sense of well-being, including being well rested, sustaining energy, reaching a state of calm, and finding hope, (2) balancing family life, including reclaiming personal sphere and nurturing relationships, and (3) managing everyday life, including keeping to the daily schedule and dealing with household chores. The integrative analysis resulted in the overarching themes of health through: (1) inner strength (confirmed), (2) recovery (expanded), (3) close relationships (confirmed), and (4) social engagements (expanded). Conclusions: The findings suggest that sleep interventions for children with ADHD and sleep problems may also be associated with positive changes in aspects of parents’ health, well-being, and family life.

## 1. Introduction

Attention-deficit/hyperactivity disorder (ADHD) is a neurodevelopmental disorder characterized by persistent patterns of inattention and/or hyperactivity–impulsivity, classified into inattentive, hyperactive–impulsive, and combined subtypes (American Psychiatric Association, 2013). Globally, ADHD affects 7.6% of 3–11-year-old children and 5.6% of 12–18-year-old adolescents (Salari et al., 2023), but it also has an impact on parents (Laugesen et al., 2016; Wiener et al., 2016) and families’ everyday lives (Laugesen et al., 2016; Mofokeng & van der Wath, 2017). Parents of children with ADHD can experience challenges and possible negative health effects, such as stress (Leitch et al., 2019), less satisfaction with leisure time, and a negative impact on health-related quality of life (Peasgood et al., 2021). In addition to the challenges posed by the ADHD diagnosis, sleep problems are common in this patient group (Bondopadhyay et al., 2022), which further contributes to the impact on family life (Gnazzo et al., 2026; Martin et al., 2019). Associations have been identified between bedtime resistance, sleep duration, and daytime sleepiness in children with ADHD and maternal mental health issues, such as anxiety, depression, and stress (Martin et al., 2021). Parents’ sleep quality and total hours of sleep are also negatively affected (Peasgood et al., 2021).

Both behavioral (Hornsey et al., 2025) and pharmacological (Larsson et al., 2022a) interventions are commonly utilized to improve sleep in children with ADHD; however, the observed effect sizes tend to range from small to moderate (Hornsey et al., 2025; Larsson et al., 2022a). Moreover, there is uncertainty about how these types of interventions impact parents’ health (Hiscock et al., 2015; Sciberras et al., 2020). In recent years, weighted blankets have been used as a non-pharmacological method to improve sleep in children with ADHD and sleep problems (Bolic Baric et al., 2021; Lönn et al., 2023b). The underlying theory is that the weight of the blankets contributes to deep pressure stimulation (Mullen et al., 2008). In children with ADHD, weighted blankets have been associated with improved sleep outcomes, including better sleep quality (Bolic Baric et al., 2021; Lönn et al., 2023b), increased total sleep time and sleep efficiency, and reduced time awake after sleep onset (Lönn et al., 2023b). By extension, the use of weighted blankets by their children could possibly also improve parents’ health and everyday life (Larsson et al., 2021); however, there is a dearth of studies in the field.

The relationship between sleep problems in children with ADHD and negative health outcomes in parents (Gnazzo et al., 2026; Martin et al., 2019, 2021; Peasgood et al., 2021) is concerning, and it is unknown whether parents’ health would improve if their child’s sleep was positively impacted by an intervention. The implementation of sleep interventions with weighted blankets among children with ADHD requires significant parental involvement, and more knowledge is needed to understand the effects and experiences of such an intervention on parents’ health and everyday life. The aim of the present study was to explore parents’ own health-related experiences as their child with ADHD and sleep problems underwent a sleep intervention with a weighted blanket.

## 2. Materials and Methods

### 2.1. Study Design

A convergent mixed methods design was undertaken to explore the aim. The four steps of convergent mixed methods design are: (1) concurrent but separate design and data collection of quantitative and qualitative data; (2) separate analyses of quantitative and qualitative data; (3) a merging of quantitative and qualitative results by identifying shared concepts and contrasts across the results; and (4) interpretation and discussion of merged results (Creswell & Plano Clark, 2017). The quantitative data are presented in accordance with guidelines found in STrengthening the Reporting of OBservational studies in Epidemiology (STROBE) (von Elm et al., 2007). The qualitative data are presented in accordance with the Consolidated Criteria for Reporting Qualitative Research (COREQ) (Tong et al., 2007).

### 2.2. Study Procedures

The present study is part of a randomized, placebo-controlled crossover trial involving a sleep intervention with weighted blankets for children with uncomplicated ADHD and sleep problems (Larsson et al., 2022b). Between January 2020 and January 2022, children recently diagnosed with uncomplicated ADHD (i.e., without comorbidities or social burden and according to the Diagnostic and Statistical Manual of Mental Disorders, Fifth Edition (American Psychiatric Association, 2013)), referred to a child and adolescent mental health service in southern Sweden, and screened positive for sleep problems were invited to participate in the research project together with one of their parents. After ensuring that the child was stable on medication, not undergoing melatonin treatment, and without significant comorbidities requiring pharmacological or psychosocial interventions, 93 parents with their 93 children agreed to participate. The sleep intervention included a weighted blanket and a control blanket in a randomized crossover design in which each child slept with the weighted blanket for four weeks and the control blanket for four weeks. During the final eight weeks, the child slept with either the weighted blanket or the control blanket, whichever they preferred. The weighted blankets were fiber blankets weighing 6, 8, and 10 kg. The appropriate weight was selected for each child based on sex, height, weight, ADHD subtype, and severity of sleep problems. Questionnaires addressed to parents were collected at baseline, 4-week, 8-week, and 16-week follow-up (Larsson et al., 2022b). The current study included parents with children aged 6–13 who had chosen to continue to sleep with the weighted blanket between the 8-week and 16-week follow-up. After the 16-week follow-up, 18 parents, together with three co-parents of children included in the research project, were invited and agreed to participate in semi-structured interviews. The interview sample was intentionally selected to ensure diversity in sex, age, place of residence, civil status, employment, and educational level (Larsson et al., 2022b).

The main findings from the randomized, placebo-controlled crossover trial showed that weighted blankets increased total sleep time, improved sleep efficiency, and reduced time awake after sleep onset, as measured by sleep actigraphy (Lönn et al., 2023b). Reduced sleep problems for children who used the weighted blanket were reported by both children and parents in questionnaires (Lönn et al., 2024). In interviews conducted after the weighted blanket sleep intervention, children described that they felt calmer and more secure (Lönn et al., 2023a), and parents reported improvements in their children’s well-being (Harris et al., 2022).

### 2.3. Outcome Measures

#### 2.3.1. Health Questionnaires

The EQ-5D-3L (EQ-5D) assesses health status within the domains of mobility, self-care, usual activities, pain/discomfort, and anxiety/depression, by five items. Each item corresponds to one domain, and problems within each domain are rated on a Likert scale (no problems = 1 point, some problems = 2 points, or extreme problems = 3 points). The responses were: (1) recalculated according to a UK value set into an index ranging from 0.00 to 1.00, where 1.00 corresponds to the best health, and (2) dichotomized into no problems vs. some or extreme problems and analyzed by item. A visual analog scale ranging from 0.00 to 100.00 points (worst imaginable health state–best imaginable health state) was used to assess current health state (EuroQol, 1990).

The Outcome Rating Scale (ORS) assesses well-being during the preceding week by four visual analog scales (0.00–10.00 points, worst–best). Well-being is assessed within the following domains: overall (general sense of well-being), individual (personal well-being), interpersonal (family, close relationships), and social role (work, school, friendships). The scales were: (1) analyzed separately, (2) computed into a total score ranging from 0.00 to 40.00 points (worst–best), and (3) dichotomized according to the cut-off for a total score of <25, which may indicate a need to seek clinical care (Miller et al., 2003).

The Brief Child and Family Phone Interview (BCFPI) is a screening tool for childhood psychiatric disorders that is used in child and adolescent mental health services. The questions from the subscales family comfort and parental mood were put to the parents. Family comfort involves questions about arguments with partner, worries about child, and others’ concern about the child, and questions are rated on a Likert scale ranging from best to worst (never = 1/sometimes = 2/often = 3/always = 4). Parental mood involves questions about poor appetite, problems with concentration, feelings of depression, anxious sleep, feeling low, and feeling resigned, and the frequency of these events is rated from best to worst (<1 day = 1; 1–2 days = 2; 3–4 days = 3; ≥5 days = 4) (Boyle et al., 2009; Cunningham et al., 2009).

#### 2.3.2. Interviews

Semi-structured interviews were conducted by IL, an experienced researcher, between May and September 2020. Of the 21 parents interviewed, four parents were interviewed in couples, while 17 were interviewed individually. Among those interviewed individually, two were parents of the same child. Questions such as the following were asked during the interview: “How are you and the family affected by the child’s sleep?”, “How have both your own situation and the family situation changed as your child started sleeping with a weighted blanket?”, and “How have your experiences of health in general been influenced by your child’s use of a weighted blanket?”. Follow-up questions were asked to elicit in-depth answers, such as “Could you tell me more about…?” and “What do you mean when you say…?” The questions were piloted in two interviews. No changes were made to the interview guide, and the two pilot interviews were included in the study. The interviews lasted between 41 and 64 min (median 53 min). The interviews were audio-recorded and transcribed verbatim.

### 2.4. Data Analysis

#### 2.4.1. Quantitative Analysis

Data are presented as mean and standard deviations or numbers and percentages. Paired-samples *t*-tests and McNemar tests were carried out to analyze differences between baseline and 16-week follow-up. Cohen’s d was used to measure effect sizes, with values of 0.20–0.49 representing small, 0.50–0.79 representing medium, and ≥0.80 representing large effect sizes (Cohen, 1988). IBM SPSS Statistics software v.28 (IBM Corp., Armonk, NY, USA) was used for performing analyses, and statistical significance was set at *p* < 0.05.

#### 2.4.2. Qualitative Analysis

The manifest data were analyzed by a qualitative content analysis (Graneheim et al., 2017; Graneheim & Lundman, 2004). The interviews were listened to, and the transcripts were read thoroughly several times. Narratives related to the study’s aim were extracted as “meaning units”, which were condensed and coded. The codes were compared for similarities and differences, grouped into eight subcategories and three categories. Coding and analysis of data were performed by the first (JSM) and last (IL) authors. Consensus regarding the results of the analysis was reached by all authors. The interdisciplinary research group consisted of registered nurses (PS, IL), health science researchers (JSM, JN), and a child and adolescent psychiatrist (HJ). Respectively, they have extensive experience of qualitative (PS, JN, IL), quantitative (JSM, PS, JN, HJ, IL), and mixed (PS, JN, IL) methods. The qualitative data analysis software NVivo v.1.6.2. (QRS International, London, UK) was used for manual coding, but not for analysis.

#### 2.4.3. Integrative Analysis

After separate quantitative and qualitative analyses, a seven-step integrative analysis was conducted to generate meta-inferences. (1) Knowledge-based inferences, (2) experience-based inferences, and (3) data-driven quantitative and qualitative inferences were identified. (4) All inferences were transformed into descriptive codes and linked with regard to similarities, differences, or connections in an inference association table, creating overarching themes. (5) Speculative inferences were removed (Younas et al., 2023). (6) Inferences were assessed for confirmation (consistency between quantitative and qualitative data), expansion (quantitative and qualitative data cover various aspects of a phenomenon by divergence or complementation), or disconfirmation (inconsistency between quantitative and qualitative data), before being converted to meta-inferences (Fetters et al., 2013; Younas et al., 2023). (7) Backward-working heuristics were performed to validate the findings. The integrative analysis was presented in a joint display (Younas et al., 2023).

## 3. Results

Of the 93 parents (nine men, 9.7%, and 84 women, 90.3%) recruited to the research project, 86 parents (seven men, 8.1%, and 79 women, 91.9%) completed the questionnaires, both at baseline and at the 16-week follow-up. Children of 68 (six men, 8.8%, and 62 women, 91.2%) of the 86 parents chose to continue to sleep with the weighted blanket and were included in the present study. In the qualitative part of the study, 21 parents (five men, 23.8%, and 16 women, 76.2%) were invited and agreed to participate in interviews. Sociodemographic data are presented in Table 1. To elicit additional information, the 21 parents were also asked about the perceived effect of the weighted blanket on their child’s sleep, of whom 16 (76.2%) reported a positive effect, two (9.5%) a partial effect, and three (14.3%) no effect.

### 3.1. Quantitative Results

From baseline to the 16-week follow-up, parents reported statistically significant improvements, with small effect sizes, in their scores for the EQ-5D index (0.83 ± 0.15 vs. 0.87 ± 0.13; *p* = 0.034), EQ-5D global health (72.41 ± 17.10 vs. 76.87 ± 16.31; *p* = 0.005), ORS individual (7.08 ± 2.22 vs. 7.55 ± 1.82; *p* = 0.045), and BCFPI subscale family comfort (5.62 ± 1.62 vs. 5.14 ± 1.66; *p* = 0.003). Scores for ORS overall, ORS interpersonal, ORS social role, ORS total, and BCFPI parental mood were unchanged over the intervention period (Table 2).

The results of analyses of separate domains of the EQ-5D and the categorization of the ORS total score using the < 25 cut-off remained unchanged over the intervention period. A larger proportion of parents reported no problems with pain/discomfort (55.9% vs. 66.2%; *p* = 0.092) and anxiety/depression (47.1% vs. 60.3%; *p* = 0.064) at follow-up compared to baseline (Table 3 and Table 4).

### 3.2. Qualitative Results

Parents’ health-related experiences as their child with ADHD and sleep problems underwent a sleep intervention with a weighted blanket were described as: (1) having a sense of well-being, including being well rested, sustaining energy, reaching a state of calm, and finding hope, (2) balancing family life, including reclaiming personal sphere and nurturing relationships, and (3) managing everyday life, including keeping to the daily schedule and dealing with household chores (Table 5).

#### 3.2.1. Having a Sense of Well-Being

##### Being Well Rested

Parents described that the child’s sleep directly influenced their own sleep. Not all parents experienced that the weighted blanket had impacted their child’s sleep, but those who experienced a positive effect of the weighted blanket described that their own sleep quality also improved, in terms of longer periods of uninterrupted sleep, calmer sleep, and longer sleep duration.

*He can continue to sleep in his bed [with the weighted blanket] … certainly I sleep better in my bed. That it isn’t so crowded and messy, because I’ve noticed that my sleep is incredibly affected by his jumping around, and that he gets up, that he moves a lot and it’s crowded. Then I sleep very badly, so I sleep better now …*

Parent no. 2

In addition, when the child used the weighted blanket, the parents no longer fell asleep while putting the child to bed, which improved their own sleep routines. Overall, this resulted in parents being more well rested, which contributed to feeling a sense of well-being.

*If I’ve gone up to put her to bed, it takes me two minutes to fall asleep. It’s just the way it is. I can’t stay awake. I think that it [my body] is like a weight. [Before the intervention] I was usually lying there in bed and then the [weighted] blanket came and then it became the same [feeling for her as when I was lying next to her], that it is heavy. There is something there [that substitutes me]. I think that’s how it has been.*

Parent no. 16

##### Sustaining Energy

When the child experienced a positive effect from the weighted blanket, parents felt they had more energy than before, which helped improve their sense of well-being. As the time it took to put the child to bed was reduced, the parents had more energy left in the evenings. When the child slept better, parents felt they had more energy the following day. They expressed that sleep was essential to have the energy to take care of their child during the day. In describing the situation before the intervention, or in cases where the intervention did not affect the child’s sleep, the process of putting the child to bed and helping them sleep through the night was found to be energy-consuming.

*He wants you to sit with him when he goes to sleep. It’s five minutes, so there’s absolutely no problem. But before [the intervention], you were tiptoeing around, because you didn’t know if he would wake up before you had gone to bed yourself … You knew that as soon as you put your head on the pillow, they would wake up… [After the intervention] I can probably relax more in the evenings too and that way I get some energy. … In this way, it [the weighted blanket] has probably positively influenced me, that I can get some energy in the evening. A little break.*

Parent no. 21

##### Reaching a State of Calm

Parents expressed that their stress and worry were affected by the child’s sleep problems and well-being. With good sleep, the parents experienced higher levels of patience and self-esteem, and a better mood, leading to a sense of well-being. Reasons for stress and worry included if their child’s sleep was too short, a long bedtime, resistance to going to bed, or a late bedtime, but also if their child was in conflict with their friends because of poor sleep. The intervention helped parents reach a state of calm and reduce stress and worry.

*It feels good to have a child who feels better, of course. It’s going in the right direction. It feels good. Just that he is comfortable with his [weighted] blanket because he expresses quite clearly if he doesn’t want something and there have been no discussions about whether he should have it, but he wants the [weighted] blanket. It feels good.*

Parent no. 21

##### Finding Hope

Parents expressed that the intervention helped them find hope regarding the difficulties and challenges often experienced by this patient group. Participation in the intervention was exciting, and parents were motivated to follow through. They expressed that the intervention was worth the time and effort they invested. Most parents had previously heard about the potential benefits of a weighted blanket for sleep problems, but were surprised by how well the blanket worked. Finding hope helped improve their sense of well-being.

*…but maybe I hadn’t thought… I probably hadn’t. You’ve thought about weighted blankets, but not that it would have been so magical on him. I didn’t think it would work so well.*

Parent no. 3

It was satisfying for the parents to see that their child’s sleep could be improved by a non-pharmacological method. Some parents even expressed that the blanket was preferable to medicating the child.

*If it’s that simple and manipulates sleep, then it’s great… It’s probably better than stuffing yourself with lots of medication, so if it works with a [weighted] blanket, it’s the absolute best.*

Parent no. 20

The intervention helped parents to understand the importance of their child’s sleep, and to change their previous explanatory model for the child’s poor sleep. Furthermore, the weighted blanket provided them with a tool to improve their child’s sleep.

*It feels like the toddler years lasted a very long time, but at least in the case of sleep, we’re starting to come out of them because of the weighted blanket. And I attribute it more to the weighted blanket than to maturity, because if we remove it, I know that in the end we will be right back where we were before.*

Parent no. 17

#### 3.2.2. Balancing Family Life

##### Reclaiming Personal Sphere

It was common for children to want or need their parents to embrace them as part of the bedtime routine, leaving parents feeling stuck for long periods. With the weighted blanket, parents could reclaim their personal space, helping balance family life. Some parents experienced that their child’s need for intimacy was reduced when the child used the blanket. This was expressed as shorter periods of embracement, a lower degree of physical contact, or less pressure in the embracement; therefore, the weighted blanket partly or completely substituted the embracement. This occurred both at bedtime and during the night if the parent and the child shared a bed. When unwanted physical contact during the night was reduced, one parent described that more emphasis was placed on embracement and physical contact during the morning instead.

*If we lie next to her, she falls asleep faster too. She can’t fall asleep on her own, so she needs the reassurance that someone is with her. So before we had this weighted blanket, we put one leg over her and held her quite tightly, because she wanted it, because she wanted to feel a bit embraced, so to speak. But when she has this … weighted blanket, it’s not needed in the same way. It is enough to put an arm over her, and she falls asleep.*

Parent no. 7

When parents experienced that their child fell asleep faster with the weighted blanket, this freed up time in the evenings, and allowed them to prioritize themselves and spend time on activities of their own choice, which helped balance family life. More time also enabled them to be more flexible about when they went to bed, and they did not need to feel forced to go to bed early to prepare for their child’s restless night.

*Of course you adapt it, then you tried to start several hours before so you had everything ready and it’s just a matter of going to bed on time. It’s a huge difference, knowing that he falls asleep at once and I can do what I want. I don’t need to sit up there. I don’t have to adapt to it, now that he can manage on his own.*

Parent no. 3

The weighted blanket seemed to contribute to children sleeping more deeply, which meant that parents did not have to curtain the level of noise as much during the evening. They could converse with their spouses or watch TV without headphones, which helped them feel they had reclaimed their personal sphere.

*He doesn’t like certain sounds when you sit and watch TV… He doesn’t like it when they talk on TV “The sound mustn’t be too loud because I don’t like it”. So with him falling asleep faster [with the weighted blanket], it makes things a little easier. So, we don’t disturb him, as it was before [the intervention].*

Parent no. 15

##### Nurturing Relationships

Parents described that family relationships improved when the child slept well as a result of using the weighted blanket. There were fewer conflicts and arguments, and if there were conflicts, they were more short-lived. Parents experienced better communication, harmony, and quality time with their child. There was a nicer atmosphere within the family. The weighted blanket could also help the parent balance days when the child struggles more with their symptoms. Poor sleep in the child led to more conflicts and less harmony in the relationship.

*She doesn’t fight as much with her little sister. She is much more patient… So she can even spend time with her little sister all of a sudden, because she has gotten a better night’s sleep [with the weighted blanket]. Otherwise, she just gets angry at everything her little sister does, always. But when she has slept well, it’s like ‘happy families’. Then she is very happy.*

Parent no. 10

Some parents experienced that using the weighted blanket for their child helped them strengthen their parental role or that uninterrupted sleep made them feel more like adults. This helped to nurture the relationship between parent and child and promote balance in family life. Poor sleep for both the child and the parent, however, could lead to parents abandoning their parental role and responsibility in the moment of struggle.

*We both get a little more prickly. Because I become, because I am also controlled by food and sleep. So if neither of us has slept well or eaten well, then both of us become aggressive … the thorns are outward and no one can have a discussion with the other and I can’t be an adult then, so then I also become a child … But if we’ve both slept [she with the weighted blanket] and eaten properly, then it’s extremely harmonious, if I do say so myself.*

Parent no. 1

#### 3.2.3. Managing Everyday Life

##### Keeping to the Daily Schedule

The sleep intervention was experienced as helping parents in various ways to maintain their daily schedules. During the measured week of the intervention, parents were instructed to report the time their child went to bed and maintain consistent bedtimes throughout both weekdays and weekends. This was experienced as improving sleep routines and raising their awareness of the importance of having these routines. Some parents felt the weighted blanket could even motivate the child to go to bed, whereas others said that the weighted blanket did not affect the actual time it took to get the child into bed.

*There has been a lot to keep track of, because of the reporting of time. I’ve gotten a better idea of when we should go to bed, I can say. Just to write it down, because I know she needs a lot of sleep.*

Parent no. 1

According to the parents, good sleep for the child made the morning routines run more smoothly. Some parents expressed that their child was easier to wake in the morning after sleping with the weighted blanket, which could also affect the timing of various morning activities. For children whose situation was unchanged by the intervention, poor sleep could still lead to a poor start to the morning and result in the morning routines taking more time and energy than expected. Parents experienced that the everyday routines felt calmer, which helped them keep to the daily schedule and manage everyday life. With good sleep for their child, they expressed that they could be more flexible and that it was no longer as important that every tasks or activities be performed in a certain order.

*He sleeps pretty deeply, but after some use of teddy bears and one of the cats tickling him a little, I get him up faster than before. Because, before [the intervention], he would always stay in bed for a long time and then it became stressful in the morning. Now [after the intervention], he gets up quite quickly, so it takes maybe ten minutes for him to get up compared to half an hour before.*

Parent no. 15

Children’s school attendance is part of the daily family schedule. With better sleep from the weighted blanket, some parents experienced that their child functioned better in school and received fewer time-consuming calls from the school. This helped them maintain their everyday life and made parents less worried. Other parents experienced no change in school contacts since the intervention started.

*It’s hard to say, but there aren’t very many days you get the call from the school anymore. I think I’ve gotten a call or two since she started with the weighted blanket, from the school. Before it was quite often and then it really was, we have even sat down with the school and what should we do to make her last all day.*

Parent no. 5

##### Dealing with Household Chores

Some parents experienced that their child helped more with household chores when their sleep improved with the weighted blanket. This included going to the grocery store and assisting with cooking and cleaning chores, which helped parents manage everyday life better.

*She is fantastic and helps [after sleep has improved with the use of the weighted blanket]. Can consider being a bit of a babysitter and start cooking. I mean, clean, pick things up, fix … Not because you nag, but she wants to learn things. It gives a lot of energy to everyone when she’s in that kind of mood …*

Parent no. 9

Regarding household chores, the parents mentioned that, with the weighted blanket, making the bed was manageable, albeit heavier. They also appreciated its design, which allowed the weighted blanket to be split into two lighter pieces for separate machine washing.

*No, it’s not [it’s not easy to make the bed with the weighted blanket], but I think it’s going pretty well anyway. You have to make the bed in a different way, when you have to put on the duvet cover because it is so heavy then. But I still think it works okay… We have a stair rail, so you can hang it a little over there when you pull on the duvet cover. I think it’s going well.*

Parent no. 8

### 3.3. Integrative Results

The analysis of the quantitative and qualitative data revealed two confirmed overarching themes: “health through inner strength” and “health through close relationships.” These themes were confirmed through the consistency observed between the quantitative and qualitative data. Additionally, the analysis identified two expanded overarching themes: “health through recovery” and “health through social engagements.” These expanded themes emerged from the complementary aspects covered by the quantitative and qualitative data.

In “health through inner strength”, the quantitative and qualitative results supported the notion that the parents felt better, calmer and more at ease, which contributed to inner strength, during and after the intervention. In “health through recovery”, the qualitative results highlighted that the intervention had a positive effect on parents’ sleep and energy levels, which contributed to recovery. In the quantitative results from the BCFPI, the parental mood scale and the question about anxious sleep were unchanged over the intervention period, giving some support to—but not full confirmation of—our qualitative results. In “health through close relationships”, the quantitative and qualitative results indicated that parents experienced a better family life during and after the intervention. In “health through social engagements”, the qualitative results indicated that parents experienced improvements in their social and everyday lives during and after the intervention. The quantitative results of the EQ-5D index supported the qualitative results, but the ORS social role was similar between baseline and follow-up measurements, thereby not fulfilling full confirmation of results. A joint display of quantitative, qualitative, and generated meta-inferences is presented in Table 6.

## 4. Discussion

This study contributes significantly by addressing the uncertainty in the existing literature regarding the impact of sleep interventions for children with ADHD on parental health (Hiscock et al., 2015; Sciberras et al., 2020), particularly for weighted blankets. The principal findings of the current study showed that parents reported improvements in quantitative measures of health status, well-being, and family life at the end of the sleep intervention. Qualitatively, parents reported an improved sense of well-being, including feeling well rested, sustained energy, calmness, and hope. Furthermore, enhancements were experienced in balancing family life, including reclaiming the personal sphere and nurturing relationships, as well as in managing everyday life, including keeping to the daily schedule and dealing with household chores. As the integrative result, two overarching themes emerged in the study: “health through inner strength” and “health through close relationships,” both supported by the consistency between quantitative and qualitative data. Additionally, the study revealed two expanded overarching themes, “health through recovery” and “health through social engagements,” showing complementary aspects covered by the quantitative and qualitative data. The findings indicate that a sleep intervention using weighted blankets for children with ADHD and sleep problems may positively influence parents’ health.

Parents’ inner strength, including mental health and well-being, was positively affected by the sleep intervention. Mental health issues, such as anxiety and depression, are common in parents of children with ADHD in general (Laugesen et al., 2016), and in parents of children with ADHD and sleep problems in particular (French et al., 2023; Lycett et al., 2014; Martin et al., 2021; Sciberras et al., 2023). However, the potential mediating role of improved sleep in children on parental health is unclear. During a behavioral sleep intervention for children with ADHD and sleep problems, no short-term effects on parental mental health were observed (Hiscock et al., 2015). However, longitudinal findings from the same research project indicated that parental depression was a factor that attenuated the sustained positive impact of the intervention on children’s sleep (Sciberras et al., 2020). Similar findings were evident in a prospective study on sleep problems in children with ADHD, where poor parental mental health was associated with more persistent sleep problems in children (Lycett et al., 2014). In our study, stress and worry were experienced less frequently after the sleep intervention, consistent with previous findings showing that adequate sleep for the child was associated with lower parental stress (Gissandaner et al., 2023).

In the current study, improved recovery for parents, including enhanced sleep quality and energy levels, was identified during and after the sleep intervention for their child. Previous research confirms a relationship between the child’s and the parents’ sleep problems (Bondopandhyay et al., 2024; Peasgood et al., 2021), suggesting a potentially bidirectional relationship (Bondopandhyay et al., 2024). Research describes the effects of sleep deprivation in parents of children with ADHD and sleep problems by highlighting difficulties related to concentration, cognitive ability, memory, and mood. Additionally, it underscores the challenges of insufficient recovery and energy depletion related to the child’s high attention demands (French et al., 2023). Thus, evaluating and promoting adequate sleep for parents is vital for healthcare professionals to assist parents in effectively managing their child’s ADHD.

Our findings revealed improvements in parents’ health during the sleep intervention, particularly in close relationships. The intervention positively impacted family life, fostering better relationships between parents and children, enhancing family dynamics, and promoting the development of their parental roles. Research on parents highlights that an ADHD diagnosis in children negatively impacts partner relationships (French et al., 2023; Leitch et al., 2019), a situation that is exacerbated by sleep problems, which reduce quality time between spouses (French et al., 2023). In the current study, parents felt better equipped to handle their parental responsibilities during and after the sleep intervention. Previous research indicates that parenting abilities are negatively affected by the child’s ADHD and sleep problems, often leading to feelings of inadequacy (French et al., 2023).

Parents experienced improved health in terms of social engagement during the sleep intervention for their child with ADHD. Parents experienced that the sleep intervention facilitated smoother daily routines as children were more rested in the morning. This finding is noteworthy, given that established routines and family structures are widely recognized as crucial to managing daily challenges (Laugesen et al., 2016). In the current study, parents also experienced that their child was more willing to help with household chores. Previous research has shown that parents often experience that children with ADHD do not contribute to performing household chores in a satisfactory manner (Spaulding et al., 2021). However, other research findings indicate that children with ADHD whose medication effects last beyond school hours engage more frequently in household chores than do those whose medication has more short-term effects (Park et al., 2022). This suggests that various factors may impact a child’s ability or willingness to perform household chores. In the current study, some parents experienced a reduced need for the school to contact them during and after their child’s sleep intervention with the weighted blanket. Existing research highlights the challenges parents of children with ADHD often encounter within the educational system (Laugesen et al., 2016; Mofokeng & van der Wath, 2017), where, particularly, mothers often bear the responsibility of advocating for their child, which is a time-consuming task (Laugesen et al., 2016).

### 4.1. Methodological Considerations

#### 4.1.1. Quantitative Data and Analysis

The quantitative results need to be discussed with regard to validity, reliability, and generalizability. The EQ-5D demonstrates poor-to-moderate responsiveness to clinical change (Tordrup et al., 2014). Although the change in index from baseline to follow-up was statistically significant, the effect size was small, which needs to be considered when interpreting the results. ORS was designed to detect changes in well-being following a therapy intervention (Miller et al., 2003), but as this intervention was not therapeutic or directed toward the parents, this may have impacted the results. The BCFPI consists of a battery of questions that are usually recalculated into subscales (Boyle et al., 2009; Cunningham et al., 2009). Only certain questions were included in the study, which limits the ability to use the subscales fully and, therefore, could be considered a limitation of this study. Positive trends in parents’ health over the intervention period were identified in ORS overall, ORS total score, EQ-5D pain/discomfort, and EQ-5D anxiety/depression. The sample size, however, may have been too small to detect a statistically significant change from baseline to follow-up. Another limitation is that the observed changes in parents’ health-related outcomes cannot be attributed solely to the weighted blanket intervention. Other factors, such as changes over time in the child’s symptoms, medication, family routines, or everyday life circumstances, may also have influenced the results.

The majority of parents in the study were mothers, which limits the generalizability of the quantitative results to fathers regarding health-related experiences related to the use of weighted blankets in children with ADHD. Future studies should aim for a more equal representation between mothers and fathers.

#### 4.1.2. Qualitative Data and Analysis

Qualitative research can be evaluated on the basis of trustworthiness, with criteria of credibility, dependability, confirmability, and transferability (Graneheim et al., 2017; Graneheim & Lundman, 2004; Lincoln & Guba, 1985). Credibility reflects confidence in data and analysis (Graneheim et al., 2017; Graneheim & Lundman, 2004). In the present study, 21 parents with diverse sociodemographic characteristics were interviewed, which enhances credibility. However, more mothers than fathers were interviewed, which could be seen as a limitation in terms of representation. Another limitation is that only parents whose children chose to continue sleeping with the weighted blanket between the 8-week and 16-week follow-ups were interviewed. However, not all the parents experienced a positive effect of the weighted blanket on their child’s sleep, which contributed to the variety of data collected. In the analysis, the data were discussed between the research team to reach a consensus regarding the interpretation. Dependability describes the extent to which a data or analysis process has changed over time (Graneheim et al., 2017; Graneheim & Lundman, 2004). The interviews were conducted immediately after the intervention period ended to avoid recall bias. Nevertheless, parents expressed that they sometimes forgot how the intervention had impacted them during the first few weeks. Dependability was strengthened by asking all parents the same questions and by having an experienced interviewer (IL). Confirmability describes the degree of neutrality of the data and the extent to which participants’ voices are reflected in the analysis process and in the results (Lincoln & Guba, 1985). It was ensured by detailed analysis descriptions and by the use of quotations, which showcased the variety of experiences. Transferability refers to the degree to which results can be transferred to other contexts (Graneheim et al., 2017; Graneheim & Lundman, 2004). Transferability was strengthened by the description of participant characteristics and the data collection process. Recruiting parents from a clinical setting likely supports transferability to similar populations and settings.

#### 4.1.3. Integrative Data and Analysis

It is a strength that the steps outlined by Younas et al. (2023) for the integrative analysis were followed. In the analysis, a thorough review of the quantitative and qualitative data was carried out. The research group formulated the integrative results into themes and, in consensus, assessed themes for confirmation, expansion, or disconfirmation. The integrated analysis enhanced the results and contributed to increased knowledge about parents’ health as their child participated in a sleep intervention with a weighted blanket.

Regarding the integrative analysis, there was suboptimal overlap between the quantitative measures and the qualitative subcategories of being well rested, keeping to the daily schedule, and dealing with household chores. This could be viewed as a limitation of the study, as the literature recommends using parallel concepts for quantitative and qualitative data in mixed methods designs (Creswell & Creswell, 2023; Creswell & Plano Clark, 2017).

### 4.2. Clinical Implications

To the best of our knowledge, no previous study has evaluated parents’ health quantitatively and qualitatively as their children with ADHD and sleep problems underwent a sleep intervention with weighted blankets. The findings from the current study support the assertion that sleep in children with ADHD is key to managing the challenges families face related to the diagnosis, and that improving children’s sleep is beneficial for parental health. Thus, healthcare professionals should adopt a family-centered approach when addressing sleep problems in children with ADHD, ensuring that interventions consider the well-being of the entire family (Coyne et al., 2018; Laugesen et al., 2016; Seniwati et al., 2023). For example, regular sleep assessments could be integrated into standard care practices to identify and address sleep issues early, thereby mitigating broader family challenges. It is also essential for healthcare professionals to offer tailored sleep interventions for children, as parents may prefer non-pharmacological interventions over medications (Laugesen et al., 2016).

## 5. Conclusions

This study suggests that parents may experience improvements in their own health when their child with ADHD and sleep problems undergoes a sleep intervention with weighted blankets. The qualitative findings indicated an enhanced sense of well-being and improved management of family life and everyday routines. In the integrative analysis, the themes “health through inner strength” and “health through close relationships” were supported by both quantitative and qualitative data, while “health through recovery” and “health through social engagements” were further elaborated through the qualitative findings. Overall, the results suggest that a sleep intervention with a weighted blanket targeting children’s sleep may be associated with positive health-related experiences for parents and improved family well-being. Future research should investigate the long-term impact of sleep interventions using weighted blankets on parents’ health and family dynamics to understand the sustainability of the observed improvements.

## Figures and Tables

**Table 1:** Sociodemographic data of parents and children.

**Variables**	**Parents in Quantitative Part of Study (*n* = 68)**	**Parents in Qualitative Part of Study (*n* = 21) ^1^**
Sex, *n* (%)		
Man	6 (8.8%)	5 (23.8%)
Woman	62 (91.2%)	16 (76.2%)
Age, years		
Mean ± SD	38.9 ± 5.2	38.9 ± 3.8
Median	38	39
Range	28–52	32–48
Civil status, *n* (%)		
Single	10 (14.7%)	4 (19.0%)
Spouse or partner	58 (85.3%)	17 (81%)
Birth country, *n* (%)		
Sweden	65 (95.6%)	19 (90.5%)
Other	3 (4.4%)	2 (9.5%)
Educational level, *n* (%)		
Primary school	5 (7.4%)	2 (9.5%)
Secondary school	23 (33.8%)	8 (38.1%)
University	40 (58.8%)	11 (52.4%)
Employment, *n* (%)		
Full-time	45 (66.2%)	12 (57.1%)
Part-time	16 (23.5%)	7 (33.3%)
Unemployed	0 (0.0%)	1 (4.8%)
Parental leave	3 (4.4%)	0 (0.0%)
Studying	3 (4.4%)	0 (0.0%)
Sick leave	1 (1.5%)	1 (4.8%)
**Variables**	**Children of Parents in Quantitative Part of Study** **(*n* = 68)**	**Children of Parents in Qualitative Part of Study** **(*n* = 18) ^1^**
Sex, *n* (%)		
Boy	38 (55.9%)	11 (61.1%)
Girl	30 (44.1%)	7 (38.9%)
Age, years		
Mean ± SD	9.4 ± 2.2	8.5 ± 2.1
Median	9	8
Range	6–13	6–13

^1^ Of the 21 parents interviewed, four parents were interviewed in couples, whereof two were not represented in the quantitative part of the study. The remaining 17 parents were interviewed individually. Among those interviewed individually, two were parents of the same child, whereof one of the parents was not included in the quantitative part of the study. SD, standard deviation.

**Table 2:** Results for EQ-5D, ORS, and BCFPI for all parents (*n* = 68).

Variables		Baseline	Follow-Up	*p*-Value	Effect Size
	*n*	Mean ± SD	Mean ± SD		(Cohen’s d)
EQ-5D ^1^					
Index (0.00–1.00)	68	0.83 ± 0.15	0.87 ± 0.13	0.034	0.26
Global health (0.00–100.00)	68	72.41 ± 17.10	76.87 ± 16.31	0.005	0.35
ORS ^1^					
Overall (0.00–10.00)	68	7.62 ± 1.86	7.89 ± 1.68	0.092	0.21
Individual (0.00–10.00)	68	7.08 ± 2.22	7.55 ± 1.82	0.045	0.25
Interpersonal (0.00–10.00)	68	7.58 ± 2.18	7.87 ± 2.10	0.240	0.14
Social role (0.00–10.00)	68	7.46 ± 1.99	7.62 ± 1.88	0.436	0.10
Total score (0.00–40.00)	68	29.74 ± 7.29	30.92 ± 6.79	0.068	0.22
BCFPI ^2^					
Family comfort (3.00–12.00)	65	5.62 ± 1.62	5.14 ± 1.66	0.003	0.39
Arguments with partner (1.00–4.00)	66	1.74 ± 0.73	1.61 ± 0.68	0.038	0.26
Worries about child (1.00–4.00)	68	2.28 ± 0.75	2.10 ± 0.74	0.045	0.25
Others are concerned for the child (1.00–4.00)	67	1.58 ± 0.65	1.45 ± 0.61	0.060	0.23
Parental mood (6.00–24.00)	66	10.14 ± 3.37	9.65 ± 4.01	0.260	0.14
Poor appetite (1.00–4.00)	67	1.33 ± 0.66	1.33 ± 0.66	1.000	0.00
Problems with concentration (1.00–4.00)	68	2.09 ± 0.96	1.97 ± 0.96	0.241	0.14
Felt depressed (1.00–4.00)	67	1.40 ± 0.74	1.40 ± 0.80	1.000	0.00
Anxious sleep (1.00–4.00)	68	2.03 ± 1.02	1.88 ± 1.02	0.159	0.17
Felt low (1.00–4.00)	67	1.69 ± 0.84	1.63 ± 0.92	0.626	0.06
Felt resigned (1.00–4.00)	67	1.64 ± 0.90	1.55 ± 0.84	0.471	0.09

^1^ Worst–best. ^2^ Best–worst. Results were analyzed with paired-samples *t*-tests and presented as means and standard deviations. BCFPI, the Brief Child and Family Phone Interview; EQ-5D, EQ-5D-3L; ORS, Outcome Rating Scale; SD, standard deviation.

**Table 3:** Results for EQ-5D domains and ORS cut-off score for all parents (*n* = 68).

Variables	Baseline *n* (%)	Follow-Up *n* (%)
EQ-5D		
Mobility		
No problems	66 (97.1%)	66 (97.1%)
Some or extreme problems	2 (2.9%)	2 (2.9%)
Self-care		
No problems	68 (100.0%)	68 (100.0%)
Usual activities		
No problems	59 (86.8%)	58 (85.3%)
Some or extreme problems	9 (13.2%)	10 (14.7%)
Pain/discomfort		
No problems	38 (55.9%)	45 (66.2%)
Some or extreme problems	30 (44.1%)	23 (33.8%)
Anxiety/depression		
No problems	32 (47.1%)	41 (60.3%)
Some or extreme problems	36 (52.9%)	27 (39.7%)
ORS ^1^		
Total score < 25	16 (23.5%)	10 (14.7%)
Total score ≥ 25	52 (76.5%)	58 (85.3%)

^1^ Worst–best. Results presented as number and percentage (%). EQ-5D, EQ-5D-3L; ORS, Outcome Rating Scale.

**Table 4:** Results for EQ-5D domains and ORS cut-off score for all parents (*n* = 68).

	**No Problems Baseline and Follow-Up**	**Some or Extreme Problems Baseline and No Problems Follow-Up**	**No Problems Baseline and Some or Extreme Problems Follow-Up**	**Some or Extreme Problems Baseline and Follow-Up**	***p*** **-Value**
EQ5D					
Mobility	64	2	2	0	1.000
Usual activities	56	2	3	7	1.000
Pain/discomfort	35	10	3	20	0.092
Anxiety/depression	27	14	5	22	0.064
	**Total score < 25 at baseline and follow-up**	**Total score ≥ 25 at baseline and <25 at follow-up**	**Total score < 25 at baseline and ≥25 at follow-up**	**Total score ≥ 25 at baseline and follow-up**	***p*** **-Value**
ORS ^1^					
Total score	6	4	10	48	0.180

^1^ Worst–best. Results were analyzed with McNemar tests. EQ-5D, EQ-5D-3L; ORS, Outcome Rating Scale.

**Table 5:** Overview of subcategories and categories.

**Categories**	Having a sense of well-being	Balancing family life	Managing everyday life
**Subcategories**	Being well rested	Reclaiming personal sphere	Keeping to the daily schedule
	Sustaining energy	Nurturing relationships	Dealing with household chores
	Reaching a state of calm		
	Finding hope		

**Table 6:** Joint display of final inferences and meta-inferences.

**Overarching Theme—Health Through Inner Strength**		
**Quantitative Results**	**Qualitative Results**	**Meta-Inferences**
EQ-5D index, EQ-5D global health, and ORS individual were statistically significantly improved over the intervention period. EQ-5D anxiety/depression, ORS overall, ORS total, and BCFPI parental mood were similar between baseline and follow-up measurements.	Parents expressed that their levels of stress and worry were affected by the child’s sleep problems and well-being. With good sleep the parents experienced higher levels of patience and self-esteem and a better mood. Supporting quotation: *It feels good to have a child who feels better, of course. It’s going in the right direction. It feels good. Just that he is comfortable with his [weighted] blanket because he expresses quite clearly if he doesn’t want something and there have been no discussions about whether he should have it, but he wants the [weighted] blanket. It feels good.* Parent no. 21	Confirmed: The quantitative and qualitative inferences indicate that a sleep intervention with weighted blankets for children with ADHD and sleep problems may impact positively on parents’ health and well-being. The finding is in line with the previous literature where child’s sleep problems are associated with parental mental health issues.
**Overarching Theme—Health Through Recovery**		
**Quantitative Results**	**Qualitative Results**	**Meta-Inferences**
EQ-5D index, EQ-5D global health, and ORS individual were statistically significantly improved over the intervention period. EQ-5D pain/discomfort and BCFPI parental mood were similar between baseline and follow-up measurements.	Parents sleep quality improved in terms of longer periods of uninterrupted sleep, calmer sleep, and longer sleep duration. Parents had more energy left in the evenings and could sustain energy during their sleep in the night. Parents regained the right to their own body and freedom of movement. Supporting quotation: *He can continue to sleep in his bed [with the weighted blanket]* … *certainly I sleep better in my bed. That it isn’t so crowded and messy, because I’ve noticed that my sleep is incredibly affected by his jumping around, and that he gets up, that he moves a lot and it’s crowded. Then I sleep very badly, so I sleep better now* … Parent no. 2	Expanded: The qualitative inferences indicate that a sleep intervention with weighted blankets for children with ADHD and sleep problems may impact positively on parents’ health in terms of recovery. The quantitative inferences offer some support to this statement. The finding is in line with the previous literature where child’s sleep problems are associated with poorer sleep in parents.
**Overarching Theme—Health Through Close Relationships**		
**Quantitative Results**	**Qualitative Results**	**Meta-Inferences**
BCFPI family comfort was statistically significantly improved over the intervention period. ORS interpersonal was similar between baseline and follow-up measurements.	Parents understood how to better help their child with sleep problems. Parents experienced a more harmonious family life and a positive development of their parental role. Supporting quotation: *She doesn’t fight as much with her little sister. She is much more patient… So she can even spend time with her little sister all of a sudden, because she has gotten a better night’s sleep [with the weighted blanket]. Otherwise, she just gets angry at everything her little sister does, always. But when she has slept well, it’s like ‘happy families’. Then she is very happy.* Parent no. 10	Confirmed: The quantitative and qualitative inferences indicate that a sleep intervention with weighted blankets for children with ADHD and sleep problems may impact positively on parenthood and family life. The finding is in line with previous literature which shows that child’s sleep problems impact negatively on family life.
**Overarching Theme—Health Through Social Engagements**		
**Quantitative Results**	**Qualitative Results**	**Meta-Inferences**
EQ-5D index was statistically significantly improved over the intervention period. EQ-5D usual activities and ORS social role were similar between baseline and follow-up measurements.	Parents were given more time and could prioritize themselves. Parents experienced better routines during the day and family members could work together to do household chores. Parents were in contact with the child’s school less. Supporting quotation: *She is fantastic and helps [after sleep has improved with the use of the weighted blanket]. Can consider being a bit of a babysitter and start cooking. I mean, clean, pick things up, fix* … *Not because you nag, but she wants to learn things. It gives a lot of energy to everyone when she’s in that kind of mood* … Parent no. 9	Expanded: The qualitative inferences indicate that a sleep intervention with weighted blankets for children with ADHD and sleep problems may impact positively on parents’ health in terms of their social life. The quantitative inferences offer some support to this statement. The finding is in line with the previous literature where a child’s ADHD diagnosis is associated with various challenges which impact negatively on parents’ social life.

BCFPI, the Brief Child and Family Phone Interview; EQ-5D, EQ-5D-3L; ORS, Outcome Rating Scale.

## Data Availability

The datasets generated during and/or analyzed during the current study are not publicly available due to legal and ethical restrictions surrounding participant confidentiality. De-identified excerpts of the data can be made available from the corresponding author upon reasonable request. Requests for data access may be subject to approval by the relevant ethics committee. For further information, please contact the corresponding author.

## Abbreviations

The following abbreviations are used in this manuscript:

ADHD	Attention-Deficit/Hyperactivity Disorder
BCFPI	The Brief Child and Family Phone Interview
COREQ	Consolidated Criteria for Reporting Qualitative Research
EQ-5D	EQ-5D-3L
ORS	Outcome Rating Scale
SD	Standard Deviation
STROBE	STrengthening the Reporting of OBservational studies in Epidemiology
